# Therapeutic Effects of PSL-Loaded PLGA-PEG-PLGA NPs in Allergic Contact Dermatitis Model Mice

**DOI:** 10.3390/molecules30153292

**Published:** 2025-08-06

**Authors:** Ryo Fujisawa, Ryuse Sakurai, Takeshi Oshizaka, Kenji Mori, Akiyoshi Saitoh, Issei Takeuchi, Kenji Sugibayashi

**Affiliations:** 1 Department of Medical Pharmacy, Graduate School of Pharmaceutical Sciences, Josai International University, 1 Gumyo Togane, Chiba 283-0002, Japan; 2 Faculty of Pharmaceutical Sciences, Tokyo University of Science, 2641, Yamazaki, Noda 278-8510, Japan; 3 Faculty of Pharmaceutical Sciences, Josai International University, 1 Gumyo Togane, Chiba 283-0002, Japan; 4 Faculty of Pharmacy and Pharmaceutical Sciences, Josai University, 1-1 Keyakidai Sakado, Saitama 350-0295, Japan

**Keywords:** poly(DL-lactide-*co*-glycolide)-*block*-poly(ethylene glycol)-*block*-poly(DL-lactide-*co*-glycolide) (PLGA-PEG-PLGA), nanoparticle, allergic contact dermatitis, prednisolone

## Abstract

This study focused on the poly(DL-lactide-*co*-glycolide)-*block*-poly(ethylene glycol)-*block*-poly(DL-lactide-*co*-glycolide) (PLGA-PEG-PLGA) triblock copolymer, which was recently reported as a novel material for polymeric nanoparticles to replace poly(DL-lactide-*co*-glycolide) (PLGA) as a drug carrier for prednisolone (PSL), and aimed to evaluate the efficacy of PSL-loaded PLGA-PEG-PLGA nanoparticles (NPs) against allergic contact dermatitis (ACD). PSL-loaded PLGA-PEG-PLGA NPs were prepared using the nanoprecipitation method, and their particle size distribution and mean particle size were measured using dynamic light scattering. 1-Fluoro-2,4-dinitrobenzene (DNFB) was used to create a mouse model of contact hypersensitivity (CHS). PSL-loaded PLGA-PEG-PLGA NPs were administered before sensitization with DNFB, and the therapeutic effect was evaluated by quantifying intracutaneous TNF-α and IL-4 levels suing ELISA. When PSL-loaded PLGA-PEG-PLGA NPs were administered before sensitization, TNF-α expression and IL-4 statements were significantly lower in the PSL-loaded PLGA-PEG-PLGA NP group than in the non-treated group. No significant difference was observed between the PSL-loaded PLGA-PEG-PLGA NP and PSL-loaded ointment groups, even though the steroid dose was 40 times lower than in the PSL-containing ointment. These results suggest that PSL-loaded PLGA-PEG-PLGA NPs may have a better effect in the treatment of ACD than PSL-loaded PLGA NPs.

## 1. Introduction

Skin is a route of administration that can safely and sustainably deliver therapeutic agents systemically and locally, avoiding the effects of hepatic metabolism [1]. Skin tissue is composed of the epidermis, dermis, and subcutaneous tissue, and the stratum corneum, the outermost layer of the epidermis, is an essential barrier that limits drug delivery [2]. In transdermal drug delivery, the use of chemical penetration enhancers and physical facilitation techniques have been studied to penetrate the stratum corneum, but possible skin irritation leads to an undesirable problem: the barrier function is compromised. To solve such problems, polymeric nanoparticle delivery systems that deliver therapeutic agents intradermally without damaging the skin barrier function have attracted attention [3,4].

Poly(DL-lactide-*co*-glycolide) (PLGA) has been extensively used as a biodegradable polymer for pharmaceutical applications and is known as a base material for polymeric nanoparticles. PLGA nanoparticles (NPs) have been reported to pass through the stratum corneum but not migrate into the dermis or subcutaneous tissue [5]. PLGA NPs have been applied in various medical fields as a base material for the transdermal absorption of drugs for therapeutic use [6,7,8,9]. In our previous study, we succeeded in preparing PSL-loaded PLGA NPs with an average particle size of approximately 30 nm using PLGA, and by administering the prednisolone (PSL)-loaded PLGA NPs to contact hypersensitivity (CHS) model mice, we were able to confirm the suppressive tendency of inflammation caused by allergic contact dermatitis (ACD) and the suppression of prolonged allergic symptoms [10].

Recently, poly(DL-lactide-*co*-glycolide)-*block*-poly(ethylene glycol)-*block*-poly(DL-lactide-*co*-glycolide) (PLGA-PEG-PLGA) triblock copolymers were reported as a novel material for polymeric NPs to replace PLGA. PLGA-PEG-PLGA triblock copolymers are highly safe because they degrade enzymatically and non-enzymatically into PLGA and PEG in the body, and PLGA is further degraded into water and carbon dioxide [11,12,13]. PEG is also used in cosmetics and pharmaceuticals, and its low toxicity ensures its safety [14,15,16]. PLGA-PEG-PLGA triblock copolymers have been investigated for various applications, including their use as temperature-responsive polymers as gels and microneedles for drug delivery and as micelles to improve blood retention [17,18,19,20,21,22]. In addition, PLGA-PEG-PLGA nanoparticles have been reported to be distributed in the epidermis and hair follicles. [23]. This report suggests that PLGA-PEG-PLGA NPs have better skin penetration and drug delivery to deeper skin layers than PLGA NPs, which infers the usefulness of PLGA-PEG-PLGA NPs. We considered that the PLGA-PEG-PLGA triblock copolymer, which can deliver drugs deeper into the skin to significantly suppress inflammation caused by sensitization to allergens that cause allergic contact dermatitis, would be useful as a base material for nanoparticles in this study.

Therefore, in this study, we planned to use PLGA-PEG-PLGA NPs as drug carriers for prednisolone. PSL-loaded PLGA-PEG-PLGA NPs were prepared and the particle size distribution of the nanoparticles and the content of PSLs were evaluated. We also evaluated the stability of PLGA-PEG-PLGA NPs by conducting stability tests at 4 °C in a cool and dark place and at 32 °C, which is the temperature of the skin surface [24], respectively. In addition, release tests were conducted at 32 °C to evaluate the release behavior of PSL from PSL-loaded PLGA-PEG-PLGA NPs. The efficacy of PSL-loaded PLGA-PEG-PLGA NPs against ACD was investigated by administering PSL-loaded PLGA-PEG-PLGA NPs to CHS model mice and observing the behavior of TNF-α and IL-4. It has been shown that CHS model mice appropriately reflect the glucocorticoid-sensitive ACD disease state and respond to chronic inflammation and cytokine activity. As such, they are useful in the study of ACD mechanisms and potential therapeutic interventions [25,26,27,28].

## 2. Results

### 2.1. Characterization of PSL-Loaded PLGA-PEG-PLGA NPs

#### 2.1.1. Properties of PSL-Loaded PLGA-PEG-PLGA NPs

**Figure 1:**
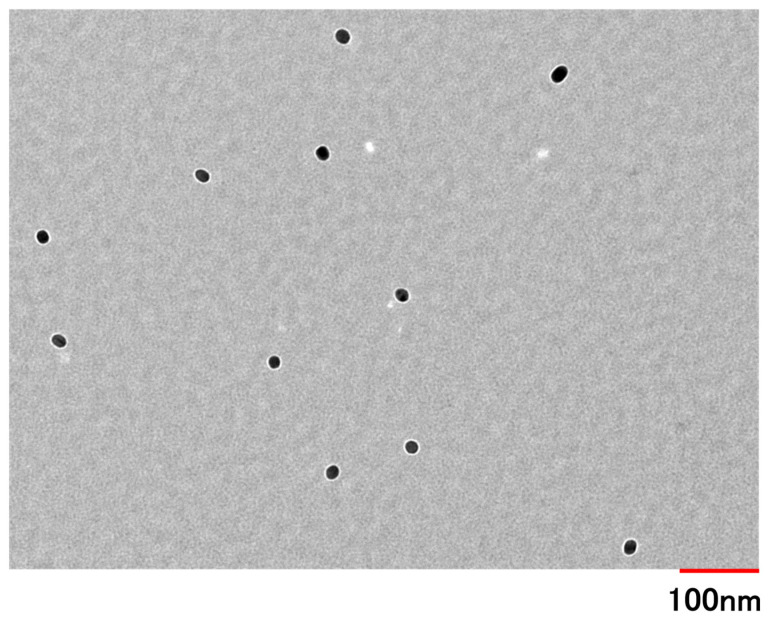
The TEM image of PSL-loaded PLGA NPs was made at an accelerating voltage of 100 kV (magnification: 20,000×).

**Figure 2:**
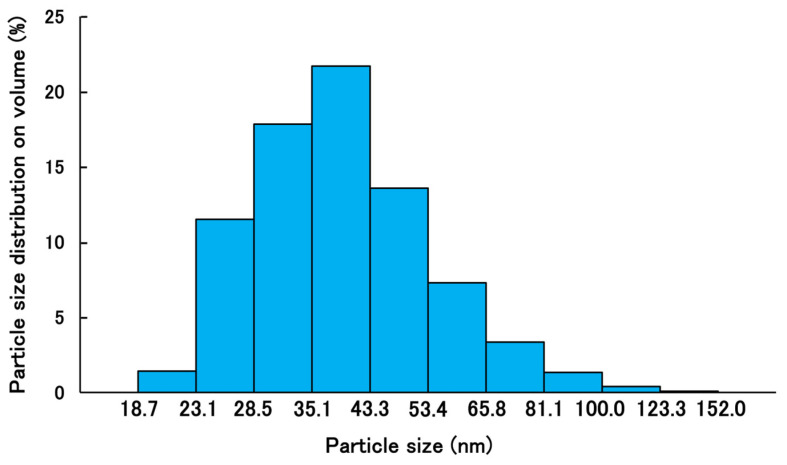
Volume size distribution of PSL-loaded PLGA-PEG-PLGA NPs by dynamic light scattering (*n* = 3).

The mean volume diameter, the loading capacity, the entrapment efficiency, the PDI, and the ζ-potential of PSL-loaded PLGA-PEG-PLGA NPs were 45.8 ± 16.5 nm, 1.4%, 24%, 0.147, and −3.98 ± 2.43 mV, respectively. The particle size distribution and TEM images of PSL-loaded PLGA-PEG-PLGA nanoparticles are shown in Figure 1 and Figure 2. When the 11 nanoparticles in Figure 1 were measured using Image J (Ver. 1.54p) [29], the particle diameter was 27.9 ± 3.5 nm. PSL-loaded PLGA-PEG-PLGA nanoparticles were spherical and dispersed without agglomeration.

#### 2.1.2. Stability of PSL-Loaded PLGA-PEG-PLGA NPs

**Figure 3:**
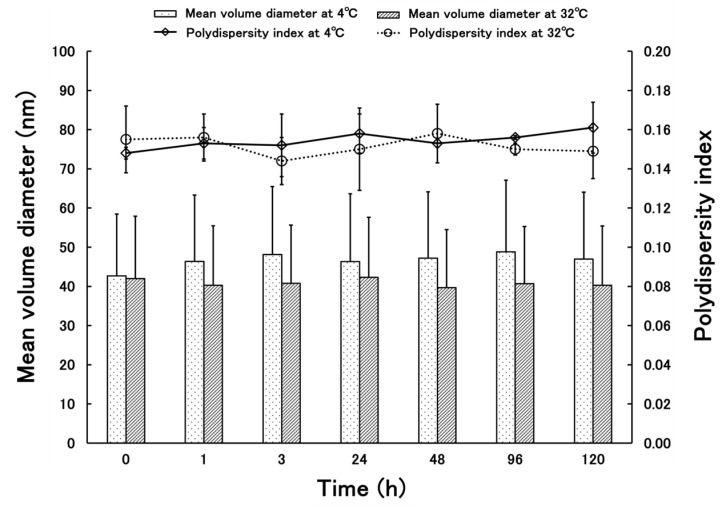
Time-department changes in mean volume diameter and polydispersity indexes of PSL-loaded PLGA-PEG-PLGA NPs at 4 and 32 °C (*n* = 3, mean ± S.D.).

Figure 3 shows the results of the stability test of PSL-loaded PLGA-PEG-PLGA NPs: At 4 °C, the mean volume diameter ranged from 42.70 to 48.83 nm and the standard deviation ranged from 15.77–18.24, indicating stability without aggregation. The PDI was 0.148–0.161, proving that the data were obtained without any variation. At 32 °C, the mean volume diameter ranged from 39.68 to 42.31 nm, and the standard deviation ranged from 14.81 to 15.91, indicating stability without agglomeration. The PDI was 0.144–0.158, proving that the data were obtained without any variation.

#### 2.1.3. Release Kinetics of PSL-Loaded PLGA-PEG-PLGA NPs

**Figure 4:**
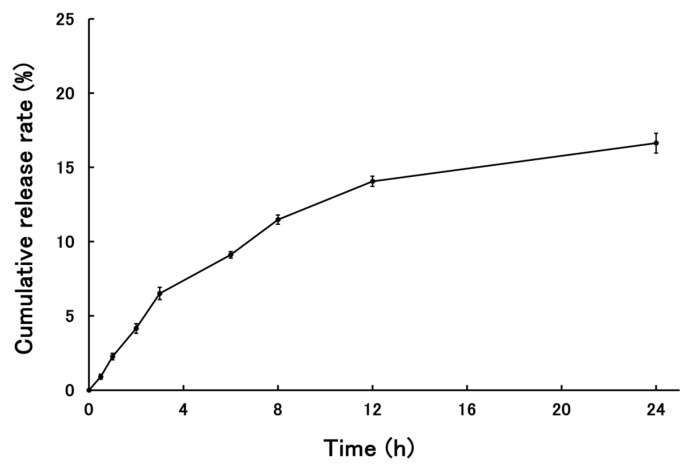
The cumulative release rate of PSL from PSL-loaded PLGA-PEG-PLGA NPs (*n* = 3, mean ± S.D.).

The release behavior of PSL from PSL-loaded PLGA-PEG-PLGA NPs is shown in Figure 4. The cumulative release rate of PSL from the nanoparticles 24 h after the start of the release test was 18.94 ± 0.73%. The release of PSL from NPs plateaued after 24 h. Data were collected up to 48 h, but since the skin was removed after 24 h and cytokine measurements were performed, only data up to 24 h are shown here.

### 2.2. Evaluation of Therapeutic Effects of PSL-Loaded PLGA-PEG-PLGA NPs Using CHS Model Mice

#### PSL Administered 2 h Before Sensitization

**Figure 5:**
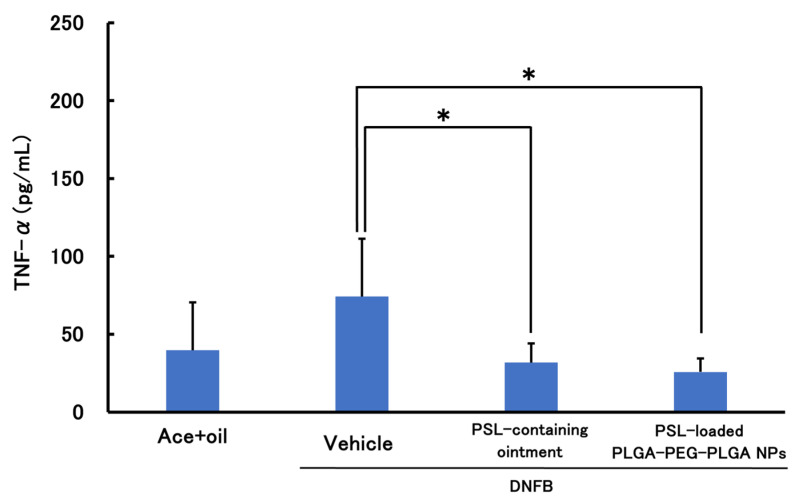
TNF-α levels in a mouse model of acute allergic contact dermatitis (*n* = 5, mean ± S.D. * *p* < 0.05 vs. DNFB, Tukey–Kramer’s test).

**Figure 6:**
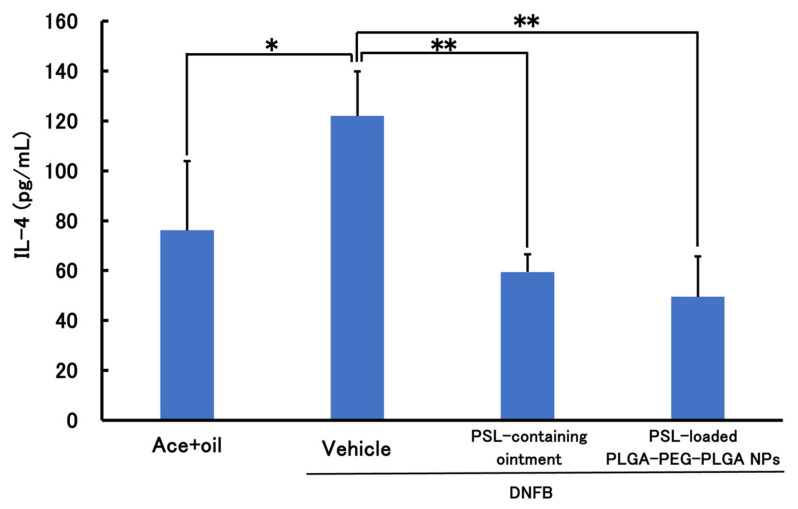
IL-4 levels in mouse models of acute allergic contact dermatitis (*n* = 3–4, mean ± S.D. * *p* < 0.05 vs. DNFB, ** *p* < 0.01 vs. DNFB, Tukey–Kramer’s test).

Figure 5 and Figure 6 show the results of the therapeutic effect of PSL-loaded PLGA-PEG-PLGA NPs on ACD when administered 2 h before sensitization. In TNF-α, a significant decrease was observed in the PSL-loaded PLGA-PEG-PLGA NPs and PSL-containing ointment groups compared to the untreated group. In IL-4, a significant decrease was observed in all groups compared to the untreated group. There were no significant differences in either TNF-a or IL-4 between the PSL-loaded PLGA-PEG-PLGA NP group and the PSL-containing ointment group.

## 3. Discussion

Comparing the present results with those reported previously, PLGA-PEG-PLGA NPs are slightly larger than the PLGA NPs. This is thought to be because the nanoparticles are slightly more hydrophilic due to the presence of PEG between the PLGAs, which allows free water to flow into the nanoparticles and increase their size. The steric hindrance in forming the nanoparticles due to the PEG entering the structure is also thought to contribute to the size [30]. The particle size measured by TEM was smaller than the particle size measured by dynamic light scattering (DLS). When measuring nanoparticles by DLS, the nanoparticles are dispersed in a solvent and swell due to the presence of water. The nanoparticles used in this study were based on PLGA-PEG-PLGA, and because they contained PEG, they were thought to be susceptible to this effect. In addition, the particle size measured by DLS is calculated based on volume. This is because it is considered more appropriate than scattering intensity criteria for nanoparticles containing drugs. There is almost no difference when calculating the particle size based on the scattering intensity standard. On the other hand, when observing nanoparticles with TEM, the nanoparticles are dried before observation, which removes moisture from the nanoparticles and causes them to shrink. As a result, it is thought that this caused the difference in particle size seen in the results of this study [31]. The drug content is higher in PLGA-PEG-PLGA nanoparticles than in PLGA nanoparticles. When nanoparticles are prepared using the same base material, it has been confirmed that larger particles tend to contain more of the drug [32,33]. However, in this case, the base material has changed from PLGA to PLGA-PEG-PLGA, so the properties of the nanoparticles are different, and it is not possible to apply the same trend. PLGA nanoparticles lack a hydrophilic structure in the base material, making it difficult for free water to enter the nanoparticles. In contrast, PLGA-PEG-PLGA contains PEG between the layers, which is hydrophilic, allowing free water to enter the nanoparticles. In fact, there are reports of cases where the structure of the nanoparticles themselves changes due to water binding to them [34]. Stability tests showed that the nanoparticles did not agglomerate even after standing for 5 days, indicating that the nanoparticles could be stored stably while maintaining their shape. It can be inferred that PLGA-PEG-PLGA NPs, like PLGA NPs, have a low risk of aggregation during administration and maintain their shape after uptake into the epidermis. The results of the release study showed that the cumulative release rate at 24 h was similar for PSL-loaded PLGA NPs and PSL-loaded PLGA-PEG-PLGA NPs. Since there was a difference in the content, we expected to see a difference in the release rate as well, but this result could be attributed to the influx of free water into the nanoparticles. In addition, although purified water was used as the external fluid in this release test, we believe that using PBS solution or physiological saline solution to simulate the biological environment would yield results closer to the release behavior upon administration. PSL is a hydrophobic drug, and the influx of free water into the nanoparticles may have acted as a barrier to release, preventing PSL from being released smoothly from the nanoparticles. Based on the evaluation of the physical properties of PLGA-PEG-PLGA NPs, PSL-loaded PLGA-PEG-PLGA NPs may be more useful. The nanoparticles are about 50 nm in size, which is not large enough to cause problems with stratum corneum penetration, and the risk of aggregation is less than with PSL-loaded PLGA NPs because the particles are not too small. Furthermore, the use of the amphiphilic PLGA-PEG-PLGA copolymer is thought to fully demonstrate the advantages of both the hydrophilic and hydrophobic properties in the nanoparticles, making them easier to use as nanoparticles.

Results of treatment experiments showed that TNF-α and IL-4 were significantly reduced when PSL-loaded PLGA-PEG-PLGA NPs were administered to CHS model mice 2 h before sensitization. This suggests the suppression of inflammation and allergic symptoms in ACD; when PSL-loaded PLGA NPs were administered under the same conditions, IL-4 was significantly reduced, confirming that allergic symptoms were suppressed. However, although TNF-α showed a decreasing trend, no significant difference could be confirmed. Furthermore, there was no significant difference between the PSL-loaded PLGA-PEG-PLGA NPs and PSL-loaded ointment groups, even though the steroid dose was 40 times lower than in the PSL-containing ointment. This is because encapsulating PSL in nanoparticles makes it possible to deliver PSL deeper into the skin than ointments, and in addition to the PSL released from the nanoparticles, PSL is also released when the nanoparticles break down through hydrolysis, suggesting that the nanoparticles may have sustained release properties and can exert a localized effect. Therefore, it was confirmed that the change of the nanoparticle base material from PLGA to PLGA-PEG-PLGA improved the tendency to suppress inflammation. Some reports suggest that PLGA-PEG-PLGA may have anti-inflammatory properties [35], which may have influenced the present results. This report states that administration of PLGA-PEG-PLGA hydrogel did not cause immune reactions such as infiltrating inflammatory cells and neovascularization, and it is thought that PLGA-PEG-PLGA nanoparticles may have the same effect when hydrolyzed in the skin.

Based on the present results, PSL-loaded PLGA-PEG-PLGA NPs showed a better therapeutic effect than PSL-loaded PLGA NPs. Both suppressed the prolongation of allergic symptoms, but PSL-loaded PLGA-PEG-PLGA NPs significantly suppressed inflammation, suggesting that they have an immediate effect. Suppression of current symptoms in addition to shortening the duration of treatment is important for symptom control. Since the skin surface is hydrophobic but the inside of the skin is hydrophilic, it is thought that PSL-loaded PLGA-PEG-PLGA NPs, which are believed to contain water, release PSL in areas closer to the inflammation, suggesting that PLGA-PEG-PLGA NPs reach deep into the skin. To confirm these findings, it is necessary to conduct release tests using model mouse skin and staining skin sections for observation.

## 4. Materials and Methods

### 4.1. Materials

PLGA-PEG-PLGA (PLGA-PEG-PLGA1005, Mw: 4000/1000/4000, monomer composition of DL-lactic acid/glycolic acid/ethylene oxide = 63/20/17) was donated by Taki Chemical Co., Ltd. (Kakogawa, Japan). L-(+)-arginine (H_2_NC(NH)NH(CH_2_)_3_ CH(NH_2_)COOH, purity ≥ 98.0%) and PSL (C_21_H_28_O_5_, >97.0%) were purchased from Fujifilm Wako Pure Chemical Corp (Osaka, Japan). Moreover, 0.5% PSL-containing ointment was purchased from Viatris Inc. (Canonsburg, PA, USA). Isoflurane for the animal was purchased from Mylan Inc. (Pittsburgh, PA, USA). ELISA MAX Deluxe Set Mouse IL-4 and ELISA MAX Deluxe Set Mouse TNF-a were purchased from Bio Legend Corp. (San Diego, CA, USA). Other chemicals were of the highest reagent grade commercially available.

### 4.2. Preparation of PSL-Loaded PLGA NP Formulation

PSL-loaded PLGA-PEG-PLGA NPs were prepared using a nanoprecipitation method. The nanoparticles were prepared as previously reported [10,36,37,38], except that PLGA-PEG-PLGA was used instead of PLGA. Compared with PLGA, PLGA-PEG-PLGA is less soluble in acetone. Before injecting the good solvent into the poor solvent, we carefully confirmed that PLGA-PEG-PLGA was completely dissolved in acetone.

### 4.3. Evaluation of Physical Properties of PSL-Loaded PLGA-PEG-PLGA NPs

#### 4.3.1. Particle Size and Zeta Potential Measurements

The particle size distribution, mean volume diameter, and polydispersity index (PDI) of PSL-loaded PLGA-PEG-PLGA NPs were measured using a ζ-potential and particle size analyzer (ELSZneo, Otsuka Electronics Co., Ltd., Osaka, Japan) by DLS under 25 °C conditions. PSL-loaded PLGA-PEG-PLGA NPs and NaCl aq of *I* = 0.308 were mixed in the same volume, and the ζ potential was measured under 25 °C conditions. The PSL-loaded PLGA-PEG-PLGA NPs were mixed with an equal volume of an aqueous NaCl solution with an ionic strength (*I*) of 0.308 M to prepare a sample (*I* = 0.154 M), and its ζ-potential was measured at 25 °C using ELSZneo.

#### 4.3.2. Loading Capacity and Entrapment Efficiency

The amount of PSL contained in the PSL-loaded PLGA-PEG-PLGA NPs was determined using high-performance liquid chromatography (HPLC, Shimadzu Corp., Kyoto, Japan) [10]. The column was Wakosil-II 5C18 AR (4.6 × 250 mm, Fujifilm Wako Pure Chemical Corp., Osaka, Japan). The nanoparticles were obtained as a suspension. The water was removed from the suspension by freezing it overnight and then drying under reduced pressure. The sample for HPLC measurement was prepared by dissolving it in acetonitrile (ACN). The column temperature was 40 °C, and the injection volume was 10 μL. The mobile phase was purified water–ACN = 6:4, and the flow rate was 1.0 mL/min. The loading capacity and entrapment efficiency of PSL were calculated using the following equation.

Loading capacity (%) = (Amount of PSL in NPs/total NPs) × 100

Entrapment efficiency (%) = (Experimental PSL loading/Theoretical PSL loading) × 100

#### 4.3.3. Morphological Observation

The particle morphology was observed using a transmission electron microscope (TEM, H-7650, Hitachi High-Technologies Corporation, Tokyo, Japan). Hydrophilic treatment was applied to the membrane surface of the colloid support membrane fixed grid (NISSHIN EM Co., Ltd., Tokyo, Japan) by irradiating it with plasma for 15 s using a plasma ion bombardment device (PIB-10, Vacuum Device Inc., Ibaraki, Japan). The grid was then immersed in a 50% PSL-loaded PLGA-PEG-PLGA NP suspension diluted with purified water for 5 min, dried in a desiccator for 24 h, and the solvent was completely removed.

The observation conditions were 100 kV of acceleration voltage and 20,000× magnification. To confirm the stability of these PSL-loaded PLGA-PEG-PLGA NPs, the mean volume diameters and polydispersity index of these NPs were measured in the same manner as described in Section 4.3.1. The storage stability of the PSL-loaded PLGA-PEG-PLGA NPs prepared in this study was confirmed by storing them at 4 and 32 °C for five days. A temperature of 32 °C was derived from the surface temperature of the human body’s skin [24]. After 1, 3, 24, 48, 96, and 120 h, the samples were taken, and mean volume diameters and PDIs were measured using ELSZneo. To evaluate the release properties of PSL-loaded PLGA-PEG-PLGA NPs used in this study, 3 mL of PSL-loaded PLGA-PEG-PLGA NPs prepared in Section 2.2 was placed in a dialysis membrane (UC20-32, molecular weight cut off: 14,000, Sekisui Material Solutions Co., Ltd., Tokyo, Japan) and placed in a vial with 97 mL of purified water at 32 °C for 24 h with water bath shaking [39]. After 0.5, 1, 2, 3, 6, 8, 12, and 24 h, each of the 3 mL samples of the outer solution were extracted and measured using HPLC, and the release rate was calculated. The same volume of purified water was added when the outer solution was extracted, and the total volume of the outer solution was always kept at 97 mL.

### 4.4. Animal Experiment

In this study, mice were divided into four groups: an untreated group with CHS, a healthy group (control group), a transdermal PSL-loaded PLGA-PEG-PLGA NP group, and a transdermal PSL-loaded ointment group. Each group consisted of five mice, for a total of 20 mice used in the experiment. All experiments were conducted using male BALB/cCrslc strain mice (9 weeks old, Japan SLC Inc., Shizuoka, Japan) weighing 20–26 g. A one-week acclimatization period was set before sensitization began, and sensitization was initiated when the mice reached 10 weeks of age. The mice were housed in CV-type animal cages maintained at 20–25 °C under a 12-h light/dark cycle (lights on at 7:00 a.m. and off at 7:00 p.m.) and had free access to food and water. All animal experiments were conducted in accordance with protocols approved by the Ethics Committee for Animal Experimentation of Josai International University and in accordance with the Josai International University Animal Experiment Guidelines (ethical approval code: 2400012).

#### 4.4.1. Preparation of a Mouse Model of Contact Hypersensitivity (CHS)

Acute DNFB-induced CHS model mice were created by sensitizing the shaved abdominal skin on Day 0 and Day 1 with 25 μL of 0.5% 1-fluoro-2,4-dinitrobenzene (DNFB). DNFB was dissolved in a mixture of acetone and olive oil (4:1, *v*/*v*) 15 min prior to sensitization. After a 3-day interval, CHS was induced by sensitizing the shaved dorsal skin on Day 5 with 10 μL of 0.3% DNFB. The control group mice were sensitized with a mixture of acetone and olive oil (4:1, *v*/*v*) instead of DNFB. On Day 0 and 1, inhalation anesthesia with isoflurane was administered in advance. On Day 5, a mixture of medetomidine hydrochloride at 0.3 mL, midazolam at 0.8 mL, and butorphanol tartrate at 1.0 mL was prepared by adding saline to make a total of 5 mL, and anesthesia was administered by intraperitoneal injection at a dose of 0.05 mL/10 g [27,28].

#### 4.4.2. Treatment Experiments Using CHS Model Mice

In the mice prepared in Section 4.4.1, PSL-loaded PLGA-PEG-PLGA NPs and PSL-loaded ointment were administered to the mice in each treatment group two hours before sensitization. At the time of administration, the mice were sedated for 20 min using the three-drug combination anesthesia described in Section 4.4.1. PSL-loaded PLGA-PEG-PLGA NPs and PSL-loaded ointment were applied to the skin, and after 20 min, the mice were awakened by intraperitoneal administration of a solution prepared by adding saline to atipamezole hydrochloride at 0.6 mL to make 5 mL at a dose of 0.05 mL/10 g. The drug sensitization time during this period was 15 min. The administered amounts were 0.2 mg/kg of PSL for PSL-loaded PLGA-PEG-PLGA NPs and 8 mg/kg of PSL for PSL-loaded ointment. The reason for the difference in PSL dosage between the two groups was that attempting to match the PSL content of PSL-loaded PLGA-PEG-PLGA NPs to that of PSL-containing ointment would result in a large amount of nanoparticle suspension, making administration difficult. Therefore, the dosage was set at the amount that could be administered via transdermal administration.

#### 4.4.3. Evaluation of Treatment Effect by ELISA

Twenty-four hours after sensitization on Day 5, skin samples were collected, homogenized in purified water for 2 min, and centrifuged at 10,000× *g* for 20 min at 4 °C. The supernatant was then collected, and cytokines were measured using the ELISA method. Measurements were performed using a plate reader (BioTek Cytation 5, Agilent Technologies Japan, Ltd., Tokyo, Japan) to measure absorbance. The wavelengths measured were 450 nm and 570 nm. The kits used for measurement were the ELISA MAX Deluxe Set Mouse IL-4 and ELISA MAX Deluxe Set Mouse TNF-α (Bio Legend Corp., San Diego, CA, USA). TNF-α was selected as an indicator of skin inflammation [40,41], and IL-4 was selected as an indicator of prolonged allergic symptoms [42,43].

### 4.5. Statistical Analisis

All data are presented as mean ± standard deviation. Statistical significance was confirmed using Tukey’s test. Significance was defined as *p* < 0.01 and *p* < 0.05.

## 5. Conclusions

Table 1 summarizes the results of PLGA nanoparticles and PLGA-PEG-PLGA nanoparticles [10]. These results suggest that PSL-loaded PLGA-PEG-PLGA NPs have better skin penetration than PSL-loaded PLGA NPs. PSL-loaded PLGA-PEG-PLGA NPs can suppress allergic reactions and inflammation caused by ACD, shorten the duration of treatment, and reduce the dose of steroids. This will reduce the prolongation of symptoms and side effects, leading to an improvement in the quality of life of ACD patients. In the future, we will conduct skin permeability tests using the skin of CHS model mice to confirm the intradermal release behavior of PSL-loaded PLGA-PEG-PLGA NPs and observe skin sections to visually assess whether the PSL-loaded PLGA-PEG-PLGA NP group shows a tendency to suppress inflammation, such as skin thickening, compared to the untreated group. Additionally, we aim to incorporate fluorescent dyes into the nanoparticles to observe how far they penetrate into the skin. Furthermore, we plan to explore the inclusion of not only PSL but also betamethasone and hydrocortisone, which are more commonly used in clinical settings.

## Figures and Tables

**Table 1:** Comparison of data for PLGA nanoparticles and PLGA-PEG-PLGA nanoparticles.

	PLGA NPs	PLGA-PEG-PLGA NPs
**Particle size**	30.6 ± 12.0 nm	45.8 ± 16.5 nm
**Loading capacity**	0.9%	1.4%
**ζ potential**	−48.34 ± 4.14 mV	−3.98 ± 2.43 mV
**PDI**	0.170	0.147
**Emission Test**	18.45 ± 1.03% (24 h)	18.94 ± 0.73% (24 h)
**Stability Test**	No change for five days (4 °C, 32 °C)	No change for five days (4 °C, 32 °C)
**Animal Experiment**	TNF-α: decreasing trend IL-4: significant decrease	TNF-α: significant decrease IL-4: significant decrease

## Data Availability

The data presented in this study are available on request from the corresponding author.

## References

[1] Mark R.P., Robert L. (2008). Transdermal drug delivery. Nat. Biotechnol..

[2] Moser K., Kriwet K., Naik A., Kalia Y., Guy R. (2001). Passive skin penetration enhancement and its quantification in vitro. Eur. J. Pharm..

[3] Cerc G., Vierl U. (2010). Nanotechnology and the transdermal route A state of the art review and critical appraisal. J. Control. Release.

[4] Khan S., Ullah M., Saeed S., Saleh E., Kassem A., Arbi F., Wanab A., Rehman M., Rehman K., Khan D. (2024). Nanotherapeutic approaches for transdermal drug delivery systems and their biomedical applications. Eur. Polym. J..

[5] Takeuchi I., Suzuki T., Makino K. (2017). Skin permeability and transdermal delivery route of 50-nm indomethacin-loaded PLGA nanoparticles. Colloids Surf. B Biointerfaces.

[6] Mir M., Ahmed N., Rehman A. (2017). Recent applications of PLGA based nanostructures in drug delivery. Colloids Surf. B Biointerfaces.

[7] Sun L., Liu Z., Wang L., Cun D., Tong H., Yan R., Chen X., Zheng Y. (2017). Enhanced topical penetration, system exposure and anti-psoriasis activity of two particle-sized, curcumin-loaded PLGA nanoparticles in hydrogel. J. Control. Release.

[8] Zhang W., Gao J., Zhu Q., Zhang M., Ding X., Wang X., Hou X., Fan W., Ding B., Wu X. (2010). Penetration and distribution of PLGA nanoparticles in the human skin treated with microneedles. Int. J. Pharm..

[9] Jain S., Mittal A., Jain A. (2011). Enhanced Topical Delivery of Cyclosporin-A Using PLGA Nanoparticles as Carrier. Curr. Nanosci..

[10] Fujisawa R., Sakurai R., Oshizaka T., Mori K., Saitoh A., Takeuchi I., Sugibayashi K. (2024). Development of PSL-loaded PLGA nanoparticles for the treatment of allergic contact dermatitis. Colloids Interfaces.

[11] Makadia H., Siegel S. (2011). Poly Lactic-co-Glycolic Acid (PLGA) as biodegradable controlled drug delivery carrier. Polymers.

[12] Brannon-Peppas L. (1995). Recent advances on the use of biodegradable microparticles and nanoparticles in controlled drug delivery. Int. J. Pharm..

[13] Keles H., Naylor A., Clegg F., Sammom C. (2015). Investigation of factors influencing the hydrolytic degradation of single PLGA microparticles. Polym. Degrad. Stab..

[14] Simone Fishburn C. (2008). The pharmacology of PEGylation: Balancing PD with PK to generate novel therapeutics. J. Pharm. Sci..

[15] Wang Z., Ye Q., Yu S., Akhavan B. (2023). Poly Ethylene Glycol (PEG)-Based Hydrogels for Drug Delivery in Cancer Therapy: A Comprehensive Review. Adv. Healthc. Mater..

[16] D’souza A., Shegokar R. (2016). Polyethylene glycol (PEG): A versatile polymer for pharmaceutical applications. Expert Opin. Drug Deliv..

[17] Yu L., Ci T., Zhou S., Zeng W., Ding J. (2013). The thermogelling PLGA-PEG-PLGA block copolymer as a sustained release matrix of doxorubicin. Biomater. Sci..

[18] Ge J., Neofytou E., Cahill T., Beygui R., Zare R. (2012). Drug release from electric-field-responsive nanoparticles. ACS Nano.

[19] Gao Y., Ren F., Ding B., Sun N., Liu X., Ding X., Gao S. (2011). A thermo-sensitive PLGA-PEG-PLGA hydrogel for sustained release of docetaxel. J. Drug Target..

[20] Pearton M., Allender C., Brain K., Anstey A., Gateley C., Wilke N., Morrissey A., Birchall J. (2008). Gene Delivery to the Epidermal Cells of Human Skin Explants Using Microfabricated Microneedles and Hydrogel Formulations. Pharm. Res..

[21] Song Z., Feng R., Sun M., Guo C., Gao Y., Li L., Zhai G. (2011). Curcumin-loaded PLGA-PEG-PLGA triblock copolymeric micelles: Preparation, pharmacokinetics and distribution in vivo. J. Colloid Interface Sci..

[22] Jeong B., Bae Y., Kim S. (1999). Thermoreversible gelation of PEG-PLGA-PEG triblock copolymer aqueous solutions. Macromolecules.

[23] Kagawa A., Sato A., Makino K., Takeuchi I. (2024). Therapeutic Effects of 30 nm Cyclosporin A-Loaded Nanoparticles Using PLGA-PEG-PLGA Triblock Copolymer for Transdermal Delivery in Mouse Models of Psoriasis. Appl. Sci..

[24] Dipasquale D., Buono M., Kolkhorst F. (2003). Effect of Skin Temperature on the Cholinergic Sensitivity of the Human Eccrine Sweat Gland. Jpn. J. Physiol..

[25] Christensen A., Haase C. (2012). Immunological mechanisms of contact hypersensitivity in mice. APMIS.

[26] Saint-Mezard P., Krasteva M., Chavagnac C., Bosset S., Akiba H., Kehren J., Nicolas J., Berard F., Kanitakis J., Kaiserlian D. (2003). Afferent and Efferent Phases of Allergic Contact Dermatitis (ACD) Can Be Induced After a Single Skin Contact with Haptens: Evidence Using a Mouse Model of Primary ACD. J. Investig. Dermatol..

[27] Röse L., Schneider C., Stock C., Zollner T., Döcke W. (2012). Extended DNFB-induced contact hypersensitivity models display characteristics of chronic inflammatory dermatoses. Exp. Dermatol..

[28] Manresa M. (2021). Animal Models of Contact Dermatitis: 2,4-Dinitrofluorobenzene-Induced Contact Hypersensitivity. Methods Mol. Biol..

[29] Igathinathane C., Pordesimo L., Columbus E., Batchelor W., Methuku S. (2008). Shape identification and particles size distribution from basic shape parameters using ImageJ. Comput. Electron. Agric..

[30] Beletsi A., Panagi Z., Avgoustakis K. (2005). Biodistribution properties of nanoparticles based on mixtures of PLGA with PLGA-PEG diblock copolymers. Int. J. Pharm..

[31] Souza T., Ciminelli V., Mohallem N. (2016). A comparison of TEM and DLS methods to characterize size distribution of ceramic nanoparticles. J. Phys. Conf. Ser..

[32] Yu L., Zhang Z., Ding J. (2011). Influence of LA and GA Sequence in the PLGA Block on the Properties of Thermogelling PLGA-PEG-PLGA Block Copolymers. Biomacromolecules.

[33] Budhian A., Siegel S., Winey K. (2007). Haloperidol-loaded PLGA nanoparticles: Systematic study of particle size and drug content. Int. J. Pharm..

[34] Zhang H., Gilbert B., Huang F., Banfield J.F. (2003). Water-driven structure transformation in nanoparticles at room temperature. Nature.

[35] Pratoomsoot C., Tanioka H., Hori K., Kawasaki S., Kinoshita S., Tighe P., Dua H., Shakesheff K., Rose F. (2008). A thermoreversible hydrogel as a biosynthetic bandage for corneal wound repair. Biomaterials.

[36] Tomoda K., Yabuki N., Terada H., Makino K. (2014). Surfactant free preparation of PLGA nanoparticles: The combination of antisolvent diffusion with preferential solvation. Colloids Surf. A Physicochem. Eng. Asp..

[37] Takeuchi I., Kobayashi S., Hida Y., Makino K. (2017). Estradiol-loaded PLGA nanoparticles for improving low bone mineral density of cancellous bone caused by osteoporosis: Application of enhanced charged nanoparticles with iontophoresis. Colloids Surf. B Biointerfaces.

[38] Takeuchi I., Kagawa A., Makino K. (2020). Skin permeability and transdermal delivery route of 30-nm cyclosporin A-loaded nanoparticles using PLGA-PEG-PLGA triblock copolymer. Colloids Surf. A Physicochem. Eng. Asp..

[39] Takeuchi I., Makino K. (2019). Biocompatibility and effectiveness of paclitaxel-encapsulated micelle using phosphoester compounds as a carrier fir cancer trearment. Colloids Surf. B Biointerfaces.

[40] Bradley J. (2008). TNF-mediated inflammatory disease. J. Pathol..

[41] Zelová H., Hošek J. (2013). TNF-α signalling and inflammation: Interactions between old acquaintances. Inflamm. Res..

[42] Bradding P., Feather I.H., Howarth P.H., Mueller R., Roberts J.A., Britten K., Bews J.P., Hunt T.C., Okayama Y., Heusser C.H. (1992). Interleukin 4 Is Localized to and Released by Human Mast Cells. J. Exp. Med..

[43] Luzina I.G., Keegan A.D., Heller N.M., Rook G.A., Shea-Donohue T., Atamas S.P. (2012). Regulation of inflammation by interleukin-4: A review of “alternatives”. J. Leukoc. Biol..

